# Left lower lobectomy and partial preservation of segmental arteries of left upper lobe: A strategy to avoid pneumonectomy in selected cases

**DOI:** 10.1111/1759-7714.13155

**Published:** 2019-07-30

**Authors:** Dario Amore, Dino Casazza, Carlo Bergaminelli, Marcellino Cicalese, Simona Massa, Alessandro Saglia, Pasquale Imitazione, Marco Rispoli, Moana Nespoli, Carlo Curcio

**Affiliations:** ^1^ Department of Thoracic Surgery Monaldi Hospital Naples Italy; ^2^ Complex Operative Unit of Pathology, Monaldi Hospital Naples Italy; ^3^ Department of Respiratory Diseases University of Naples “Federico II”, Monaldi Hospital Naples Italy; ^4^ Department of Anesthesia and Intensive Care Monaldi Hospital Naples Italy

**Keywords:** Lung cancer, lung‐sparing resection, pulmonary artery invasion

## Abstract

In this article we report two cases of left lower lobe lung cancer undergoing a surgical procedure that allowed the preservation of lung parenchyma and avoided pneumonectomy. The first case concerned a left lower lobe non‐small cell lung cancer with extracapsular spread in a metastatic interlobar lymph node and the second a left lower lobe lung cancer with invasion of the pulmonary artery at the origin of lobar branches to the lower lobe. In both cases, a lung‐sparing surgical treatment was preferred and a left lower lobectomy was performed with division of lingular arteries and the interlobar artery, preserving the remaining arterial branches to the upper lobe.

## Introduction

The infiltration of the pulmonary artery by lung cancer and the extracapsular extension of interlobar lymph node metastasis remain one of the most challenging conditions encountered in thoracic surgery. In such situations, lung‐saving procedures are strongly advocated because not all patients are eligible for pneumonectomy and several studies in patients with N1 involvement have found no significant differences in survival in patients undergoing lobectomy compared to those undergoing pneumonectomy.^1^ We herein report a procedure that, in selected cases, allows lung parenchyma to be preserved thus avoiding resections more extensive than a lobectomy.

## Case report

### Case 1

**Figure 1:**
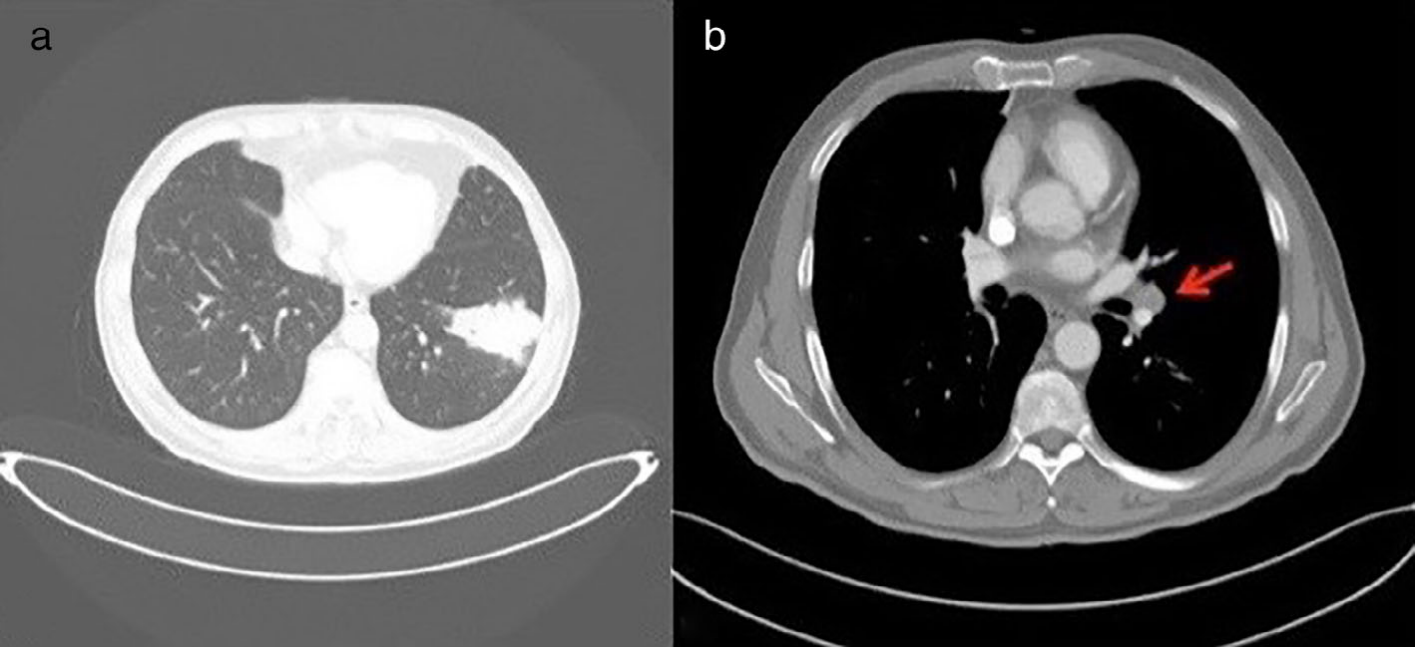
Chest computed tomography (CT) images. (**a**) Malignant lesion in the left lower lobe. (**b**) Mediastinal window revealing an enlarged interlobar lymph node (red arrow).

**Figure 2:**
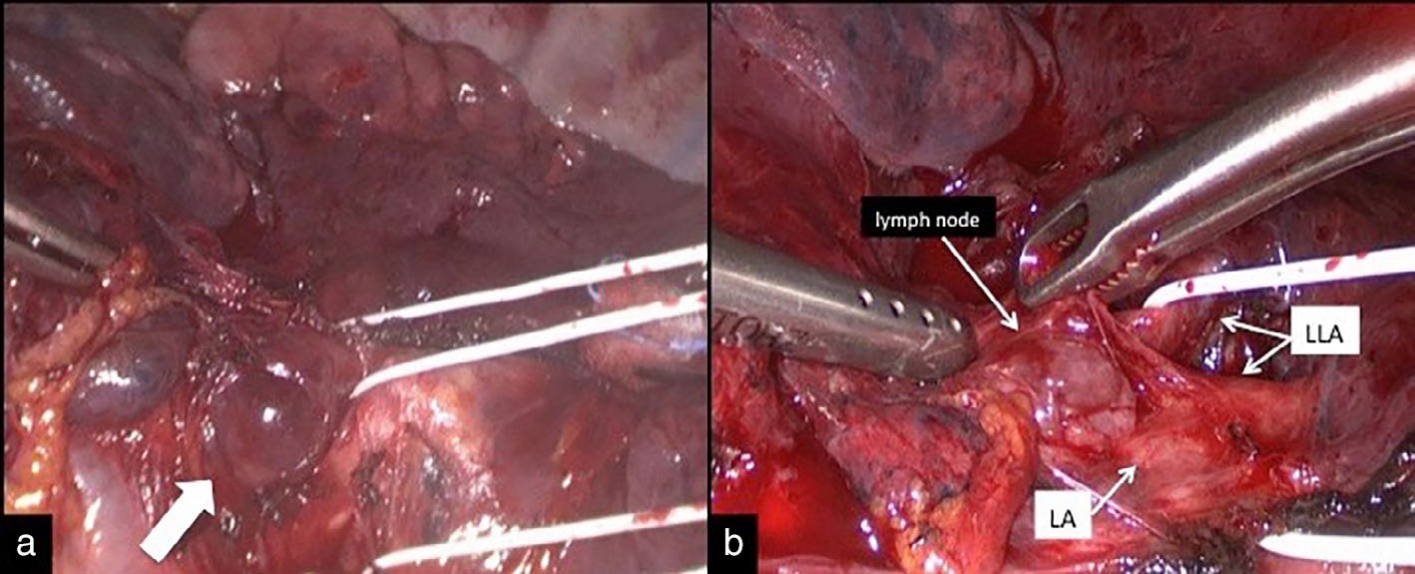
Intraoperative view after dissection of the fissure. (**a**) Enlarged lymph node in the anterior portion of the oblique fissure (white arrow). (**b**) Lingular artery infiltrated by the interlobar lymph node. LA, lingular artery; LLA, lower lobar artery.

**Figure 3:**
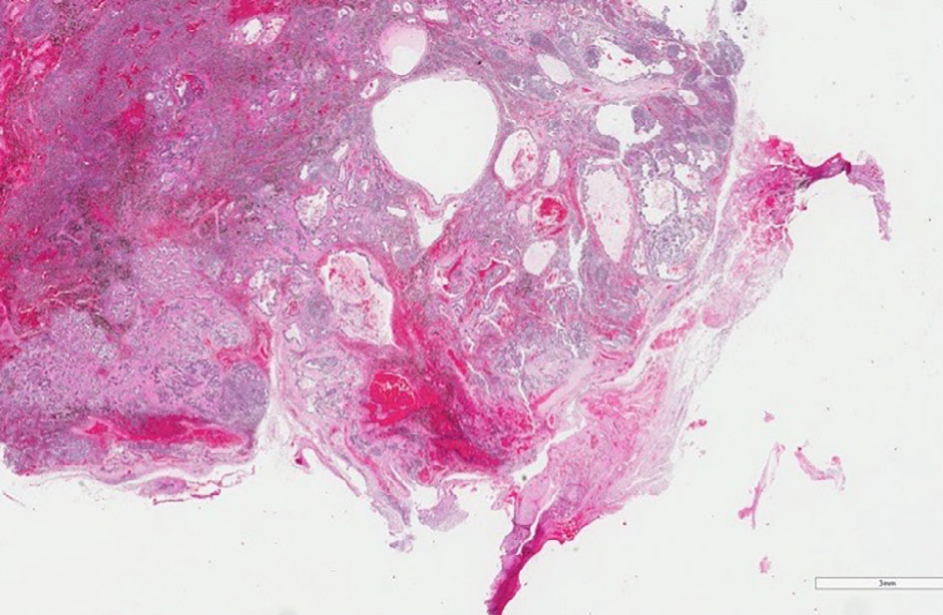
Histologic feature of lymph node metastasis with adhesion to perinodal fatty tissue (hematoxylin and eosin staining; scale bars: 3 mm).

A 72‐year‐old man, with cytologically‐proven lung adenocarcinoma, was admitted to our unit for surgical treatment. Contrast‐enhanced computed tomography (CT) scans revealed a mass measuring 60 x 40 mm in the left lower lobe and an enlarged left interlobar lymph node (Fig 1a,b). Positron emission tomography (PET) scan demonstrated focal fluorodeoxyglucose uptake in a left lower lobe lung mass, without evidence of lymphadenopathy or distant metastases; the standardized uptake value was 4.7. The patient was scheduled for thoracoscopic left lower lobectomy plus lymphadenectomy. During fissure dissection, an enlarged interlobar lymph node (station 11 lymph node), infiltrating the segmental artery for the lingula, was exposed and sampled with evidence of metastatic disease on frozen‐section examination (Fig 2a,b). Mediastinal lymph node dissection did not reveal malignancy and a left lower lobectomy was performed. After careful dissection of the lung tissue through the fissure, the segmental lingular artery, proximally free of the malignant invasion, was closed by stapler device allowing a complete removal of the interlobar lymph node metastasis. The interlobar artery was then isolated and divided with an endovascular stapling device preserving the remaining arterial branches for upper lobe (Video S1). At this point, the lobectomy was easily completed by stapling the inferior pulmonary vein and the left lower lobe bronchus. The patient had an uneventful recovery and was discharged home on postoperative day 4. Postoperative staging was pT4N1M0. Final histopathological examination revealed a moderately differentiated adenocarcinoma and extracapsular extension of lymph node metastasis to station 11 (Fig 3).

### Case 2

**Figure 4:**
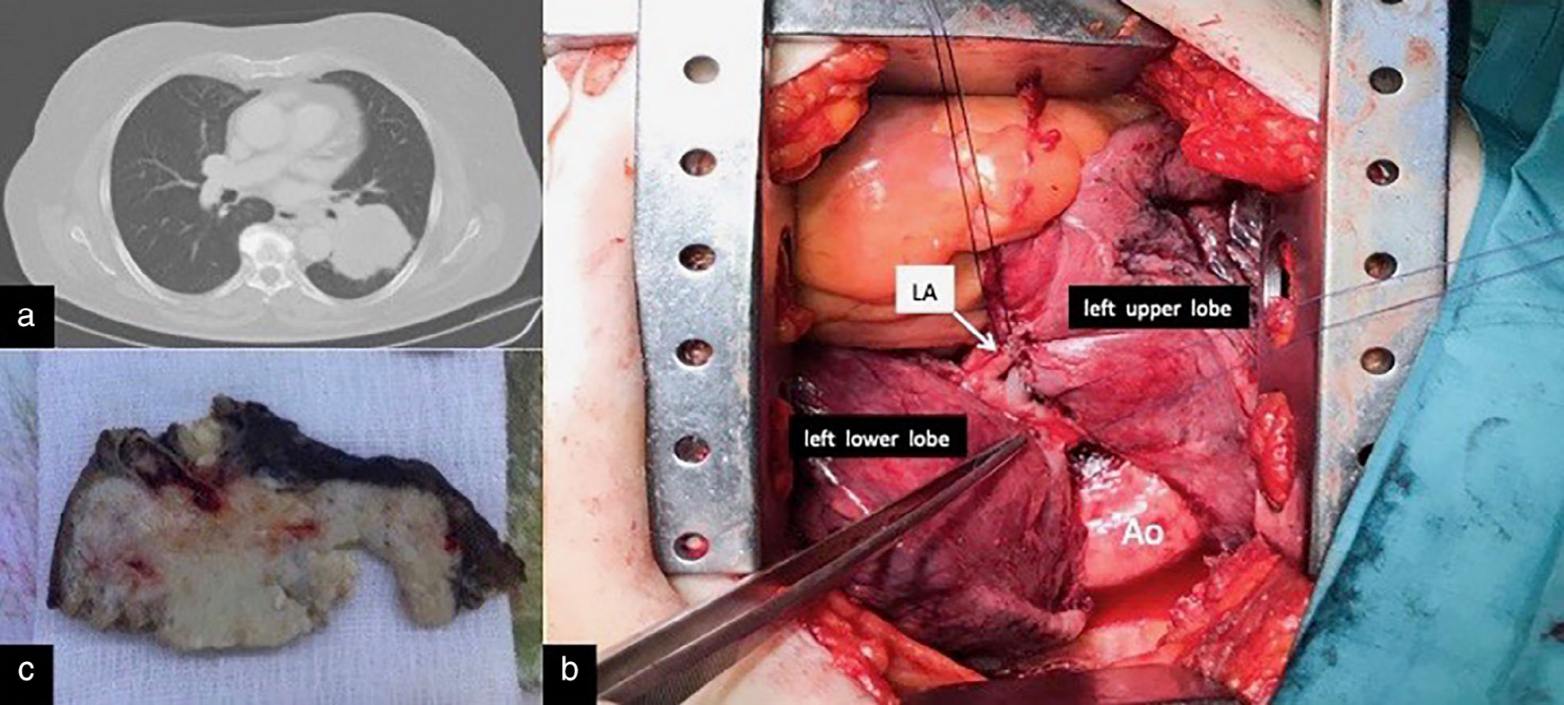
(**a**) Preoperative chest computed tomography (CT) scan in axial plane shows a left lower lobe mass. (**b**) Surgical forceps indicate infiltration of the pulmonary artery at the origin of lobar branches to left lower lobe by lung cancer. (**c**) Macroscopic appearance of resected tumor. LA, lingular arteries; Ao, aorta.

A 73‐year‐old woman was admitted to our unit for treatment of lung adenocarcinoma. Preoperative chest CT scans showed a solid mass with a maximum diameter of 60 mm in the left lower lobe (Fig 4a). PET scan demonstrated intense uptake in the left lower lobe lesion; the standardized uptake value was: 13,6. Preoperative physiologic assessment revealed predicted postoperative forced expiratory volume in the first second (PPO FEV_1_) > 60%; PPO diffusing capacity for carbon monoxide (DLCO) within 60% and 30%. The cardiopulmonary exercise test revealed a peak oxygen consumption (VO2 peak) within 10–20 mL/kg/minute. The patient, regarded as a middle risk for anatomic resection, was scheduled for a left lower lobectomy performed via a lateral thoracotomy. After a careful transfissure dissection, infiltration of the inferior aspect of the interlobar artery by the lung cancer was observed (Fig 4b). Once the viability of the lingular arteries was established, the arterial branches to the lingula and the interlobar artery were transected by stapling device preserving the remaining arterial branches to the upper lobe. The left lower lobectomy was completed with transection of the inferior pulmonary vein and the lower lobe bronchus. The procedure was followed by an uneventful course and the patient was discharged on the fifth postoperative day. At macroscopic examination, the tumor was strongly adherent to the arterial wall (Fig 4c). Postsurgical pathologic staging was T3N1M0.

## Discussion

In the last decades, some authors have demonstrated that reconstructive parenchymal‐sparing procedures provide the possibility of achieving a margin‐negative resection, avoiding postoperative morbidity and mortality associated with pneumonectomy for patients with infiltration at the origin of the lobar branches of the pulmonary artery by operable non‐small cell lung cancer (NSCLC), or for lung cancer patients with interlobar lymph node metastases.^2^ In the first case reported here, the primary tumor was located in the left lower lobe and a metastatic interlobar lymph node with extracapsular spread involved the segmental artery for the lingula. To achieve a complete removal of the interlobar lymph node metastasis, the lingular artery was sutured with a stapling device and then during left lower lobectomy, the interlobar pulmonary artery was transected, preserving the remaining arterial branches for upper lobe. In the second case, the patient had a left lower lobe cancer which invaded the pulmonary artery at the origin of lobar branches to lower lobe. Since pulmonary function tests indicated inability to tolerate pneumonectomy, a parenchymal‐sparing resection was preferred: a left lower lobectomy was performed by transection of lingular arteries and interlobar pulmonary artery, preserving the remaining arterial branches for upper lobe. Although ligation of pulmonary artery branches leads to a decreased pulmonary blood flow, several experimental studies have demonstrated that bronchial circulation responds to pulmonary ischemia through hypertrophy and development of extensive anastomoses between the enlarged bronchial system and the pulmonary arterial system in order to maintain blood flow to the lung and participate in gas exchange.^3^, ^4^ To the best of our knowledge, in the recent international literature, few cases have been reported in which the interlobar pulmonary artery, during left lower lobectomy, was sutured with only preservation of the branches supplying the anterior and apicoposterior segment of the upper lobe.^5^

## Disclosure

No authors report any conflict of interest.

## Supporting information


**Video S1.** Legend 1: In this video, during thoracoscopic left lower lobectomy, the lingular artery is encircled with a silicone vessel loop and transected with a stapler. The complete dissection of the enlarged interlobar lymph node is then followed by division of the interlobar artery with endovascular stapling.
